# Great apes are sensitive to prior reliability of an informant in a gaze following task

**DOI:** 10.1371/journal.pone.0187451

**Published:** 2017-11-22

**Authors:** Benjamin Schmid, Katja Karg, Josef Perner, Michael Tomasello

**Affiliations:** 1 Department of Developmental and Comparative Psychology, Max Planck Institute for Evolutionary Anthropology, Leipzig, Germany; 2 Department of Psychology, University of Salzburg, Salzburg, Austria; University of Portsmouth, UNITED KINGDOM

## Abstract

Social animals frequently rely on information from other individuals. This can be costly in case the other individual is mistaken or even deceptive. Human infants below 4 years of age show proficiency in their reliance on differently reliable informants. They can infer the reliability of an informant from few interactions and use that assessment in later interactions with the same informant in a different context. To explore whether great apes share that ability, in our study we confronted great apes with a reliable or unreliable informant in an object choice task, to see whether that would in a subsequent task affect their gaze following behaviour in response to the same informant. In our study, prior reliability of the informant and habituation during the gaze following task affected both great apes’ automatic gaze following response and their more deliberate response of gaze following behind barriers. As habituation is very context specific, it is unlikely that habituation in the reliability task affected the gaze following task. Rather it seems that apes employ a reliability tracking strategy that results in a general avoidance of additional information from an unreliable informant.

## Introduction

Social animals, including our own species, often rely on information from other individuals. For adult humans that means for example relying on directions offered by a local when they visit a city they are unfamiliar with. Non-human animals rely on information from others in even more serious situations: vervet monkeys for example use alarm calls to warn conspecifics from predators, they even give specific calls for different predators that resemble semantic communication [1], and bees for example do a waggle dance to tell others where to find food [2]. Some species also rely on unwittingly offered information, obtained by deducing that information from cues in other animals’ behaviour (for example when following another individual’s gaze; e.g. [3]). While most interactions with informants occur within species, there are animals that are able to interpret cues and signals even from individuals from other species, such as vervet monkeys responding to starling alarm calls [4] or apes following the gaze of a human [5].

At the same time, relying on others can be costly as they may provide inaccurate information. When animals leave a food source relying on an alarm call announcing a predator, this will possibly result in losing access to that food source. This loss is evidently justifiable, if there is actually a predator around. However, as is obvious not least by many videos shared on the internet by pet owners, animals are prone to accidental mistakes. If the source of information in our previous example, the individual giving the alarm call, mistakenly uttered that alarm call with no predator around, relying on it will make the other animal lose their food source for no gain. In fact, some species have even adapted their behaviour to exploit flight responses to their alarm calls and engage in deceptive alarm calls [6]. It is therefore necessary for animals to adapt their behaviour according to the reliability of their source of information, in order to avoid error and exploitation.

One such adaption can be seen in habituation to repeatedly uttered false information, for example to unjustified alarm calls when there was no predator around [7]. Yet habituation is rather inflexible as it is restricted to the specific circumstances that the animal habituated to, and will not be exhibited in a conceptually different situation. In a study with vervet monkeys, Cheney and Seyfarth [7] found that only if the content of information shared by the informant is highly similar in both situations (e.g. two different vocalisations that more or less both express excitement about the encounter of another group of conspecifics), habituation can be transferred across calls by the same informant. In contrast, when the content of the information only slightly differs, habituation is not transferred. Monkeys that habituated to an unjustified leopard alarm call show an uninterrupted response when they hear a bird alarm from the same individual.

Adult humans often believe they can judge the reliability of another individual already from a first impression, and even children change their behaviour after just one utterance of false information [8]. Such rapid adaption is unlikely to be caused by mere habituation. Among adult humans and human children, also dogs show a more efficient way to deal with false information, than would be expected from mere habituation. They can assess the reliability of an informant based on few prior observations of reliable or unreliable behaviour and adapt their own behaviour accordingly. Domestic dogs quickly cease to follow pointing gestures by an unreliable informant in an object choice task, while still following gestures of a naïve informant in the same task [9]. Initially exhibiting trust in the experimenter, the dogs in this study adjusted their behaviour after only two occasions where the experimenter offered inaccurate information.

Human children, already below 4 years of age, are even more proficient in their assessment of an informant’s reliability. A large body of research shows that children preferably rely on information from a person that was accurate in a previous task as compared to a previously inaccurate person (for a review see e.g. [10]). Children discriminate between a reliable and an unreliable informant by comparing their testimony to their own experience [11]. This lets them endorse labels for novel objects from a person that had labelled know objects accurately over concurring labels by an inaccurate labeller [12]. They even seem to rely more on prior reliability as an indicator of whether an individual’s testimony should be trusted than on other features such as age [13] or accent [14]. In a study by Rakoczy, Warneken and Tomasello [15] children at 4 and 5 years of age preferably learned the rules for a novel game from a puppet that was previously reliable in a labeling task as well as other nonverbal tasks over an unreliable puppet. These studies show that children possess a more flexible way to deal with false information than mere habituation. They can infer the reliability of an informant in one task, and use that information to adjust their behaviour in a separate, subsequent task with the same informant, even if that other task is conceptually quite different.

Similarly, Chow, Poulin-Dubois and Lewis [16] found that the reliability of a person’s emotional signals influenced whether 14-month old infants followed the experimenters gaze behind a barrier. In their study, they confronted human infants with a reliable or an unreliable informant. Reliable meant that this person expressed joy over a toy in a container, while the unreliable person expressed joy over an empty container. In a subsequent gaze following task, the infant’s view was occluded from what the experimenter gazed at by a barrier. When the experimenter was unreliable in the previous emotion expression task, the infants were less likely to follow the experimenter’s gaze, which meant to crawl to a position where their view was not obstructed by the barrier. This was likely not due to a general avoidance of the unreliable informant, as when the differently reliable informants gazed at an object in front of the barrier, children followed both informants equally likely. Rather it seems that the infants inferred the reliability of the informant in the first task, and deliberately applied that judgement when confronted with more information from the same individual in the second task.

Like humans, great apes are highly social animals that could well benefit from evaluating informants’ reliability. To explore the evolutionary roots of human children’s proficiency in selective trust in informants, our goal was to investigate whether prior reliability of an informant in one task affected great ape’s behaviour in a subsequent, conceptually different task where they needed to rely on the same informant. In the study by Chow et al. [16], 14-month-old human infants followed the gaze of an experimenter less readily, when this experimenter was unreliable in a previous task compared to when the experimenter was reliable. We considered this paradigm a suitable method for our study, as great apes have been shown to follow the gaze of another individual to a similar extent as human children (for a comparison of both species’ gaze following behaviour see [5]). Furthermore, apes do not just automatically respond to gaze direction, but are able to understand gaze as referential information. They do follow gaze to a target out of their initial line of sight behind a barrier [17, 18], but they do not follow the gaze of an experimenter when that experimenter does not have perceptual access to a target [19]. To establish the informant prior to our gaze following task as reliable or unreliable we adapted a method where the experimenter gives accurate or inaccurate cues about the location of a hidden object, similar to the object choice task used by Takaoka et al. in their study with dogs [9].

As the two tasks are conceptually quite different, we would not expect habituation in the object choice task to affect gaze following in a subsequent task. Rather if the gaze task is affected by the prior object choice task, we assume that apes were able to infer the reliability of the informant in the object choice task and track this reliability across situations.

We used a design similar to the studies by Schofield and Behrend [8] and Takaoka et al. [9] to investigate how great apes react to changes in the reliability of an informant. When repeatedly confronted with the same individual in a gaze following task, habituation during this task predicts apes gaze following to decrease over time. This can be seen in existing gaze following studies with great apes [17, 20]. Similarly, when apes are confronted with an informant that is reliable first and unreliable later, we would expect a decrease in gaze following based on an assessment of reliability [8, 9]. However, when apes are confronted with an informant that is unreliable first and reliable later, we would expect an increase in gaze following if animals considered only the informant’s reliability.

Additionally, we wanted to investigate processes underlying such selectivity. Similar to Chow et al. [16], we wanted to compare the influence of prior reliability on a rather automatic gaze following process and a more deliberate gaze following process. This can help discern a general avoidance and a more explicit process underlying the selectivity.

Animals need to be selective in whether or not to rely on information offered by other individuals or deduced from their behaviour, as this information might be inaccurate. Habituation is a rather inflexible response to inaccurate information. In highly social species that encounter a lot of cues and signals in frequent interactions with differently reliable informants we might find evolutionary roots of the ability we see in human children to assess the reliability of a source of information and adjust their behaviour accordingly even in conceptually different situations. Thus in our study, we confronted great apes with a reliable or unreliable informant in an object choice task to see whether that would in a subsequent task affect their gaze following behaviour in response to the same informant.

## Method

### Participants

Participants in our study were 16 chimpanzees, 7 bonobos, 4 gorillas and 6 orang-utans from Leipzig Zoo, Germany. The apes are all housed at the Wolfgang Köhler Primate Research Center within the zoo. This facility offers a total of 12210 m^2^ in outdoor enclosures and 1307 m^2^ in indoor enclosures. Various trees, platforms and ropes provide spaces for climbing. In a concerted effort by both zoo keepers and researchers, the apes receive enrichment in form of various foraging tasks on an almost daily basis. The research was conducted in accordance with the governing laws and regulations concerning research with animals in Germany and was approved by the local animal ethics committee, the Köhler Primate Center Animal Research Committee. Our subjects were never food deprived and water was available ad libitum. They could choose to stop participating at any time. The subjects were all tested by the same male experimenter whom they were familiar with for several months.

### Design

Each subject received both the reliable and the unreliable condition. A first group of subjects (*N* = 18) experienced the experimenter to be reliable first, for the other group (*N* = 15) the experimenter was unreliable first. The unequal sample sizes are due to a criterion we used in the reliability task, which led to one dropout in the “reliable first” group compared to three dropouts in the “unreliable first” group (more details on criterion below). Order of conditions was counterbalanced within species. Per condition, two consecutive sessions were conducted per subject resulting in two sessions with the experimenter being reliable followed by two sessions with him being unreliable or vice versa. Each session was conducted on a different day and consisted of 8 trials of the reliability task followed by 8 trials of the gaze following task.

### Reliability task

**Fig 1:**
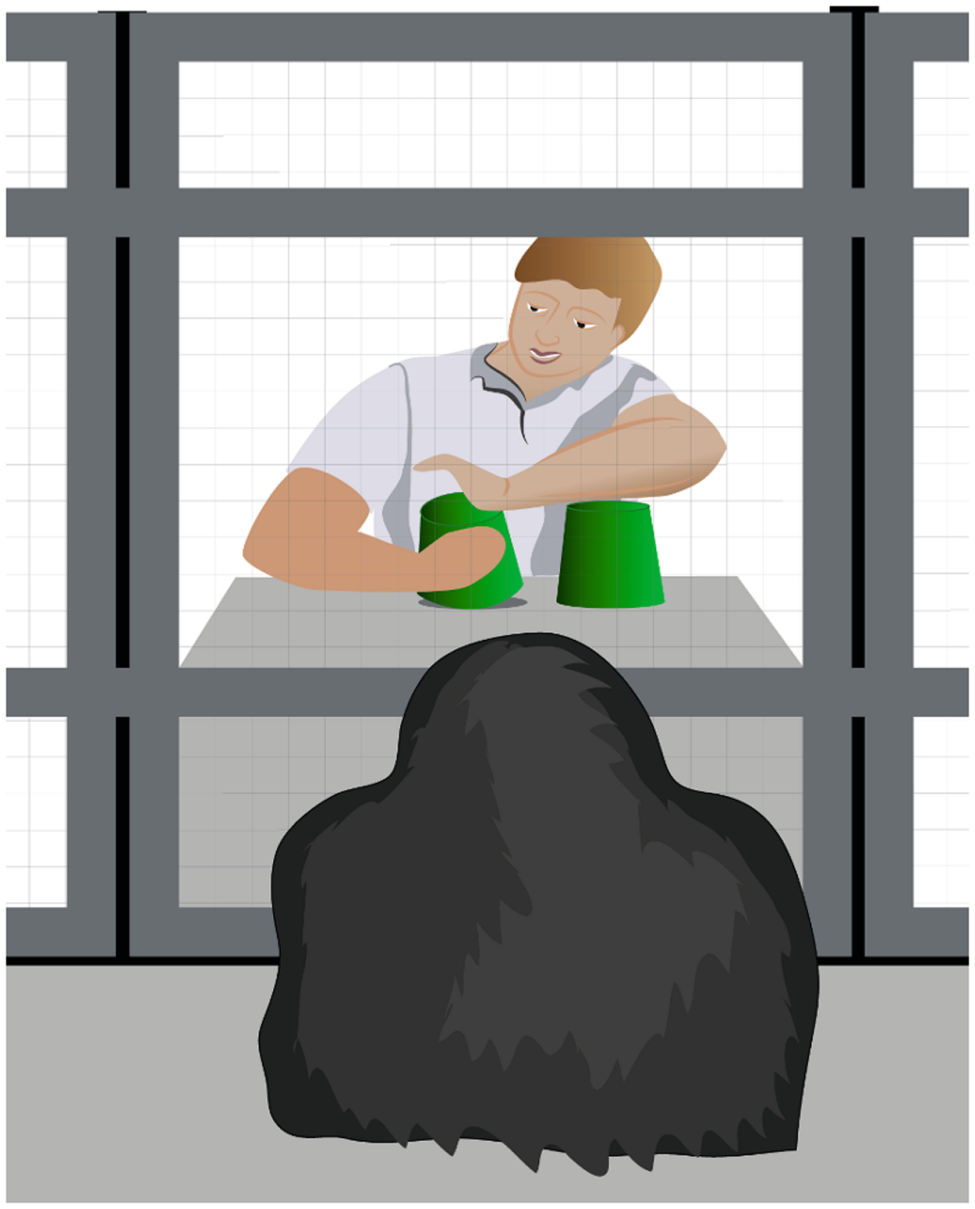
Setup for the reliability task.

To establish the experimenter as reliable or unreliable we adapted a method where the experimenter gives accurate or inaccurate cues about the location of a hidden object (e.g. [21]). In our adaption of this task, the experimenter hid a food reward behind an occluder under one of two cups, and then produced a vocal hint (“mmh” followed by a sound supposedly resembling a chimpanzee’s food call) while looking under one of the two cups (see Fig 1) that either contained the hidden reward (reliable condition) or was empty (unreliable condition).The ape was then allowed to point to one of the two cups and the content of both cups was revealed. When the ape had picked the cup containing the reward, it got fed the reward right away. When it had picked the empty cup, the reward was removed from the tray and fed to the subject after a brief delay trough the mesh right beside the setup. In the first session of either condition, the subjects had to pass a criterion before we continued with the gaze following task. This was to ensure that they were paying attention to the reliability of the experimenter’s cues. In the reliable condition, when paying attention and treating the cues as referential, the ape should choose the cued cup in the majority of trials. In the unreliable condition in contrast, the ape should not rely on the experimenter’s cues and should be guessing instead. The criteria for inclusion was as follows: For the reliable condition apes were expected to follow the cues in 6 out of 8 trials, and for the unreliable condition apes were expected to follow the cues in more than 2 but no more than 6 out of 8 trials. If the subjects passed this criterion after 8 trials, we immediately continued with 8 trials of the gaze following task. In the next session of the same condition, apes did perform the reliability task again, but did not have to pass the criterion a second time. If the subjects did not pass the criterion after 8 trials, we did not continue with the gaze following task, but rather repeated the reliability task on a following day, for up to 8 sessions. If the criterion had not been passed in the eighth session, the subject would drop out of the study. This lead to one dropout in the reliable condition in the “reliable first”- group due to a side bias of one bonobo, and three dropouts in the unreliable condition in the “unreliable first”- group as three chimpanzees continued to follow the cues of the experimenter for more than 6 trials per session even in the eighth session. The remaining subjects passed the criterion in the reliable condition on average in the 1.73th session, in the unreliable condition on average in the 1.91th session (for more information see S1 Text).

### Gaze following task

**Fig 2:**
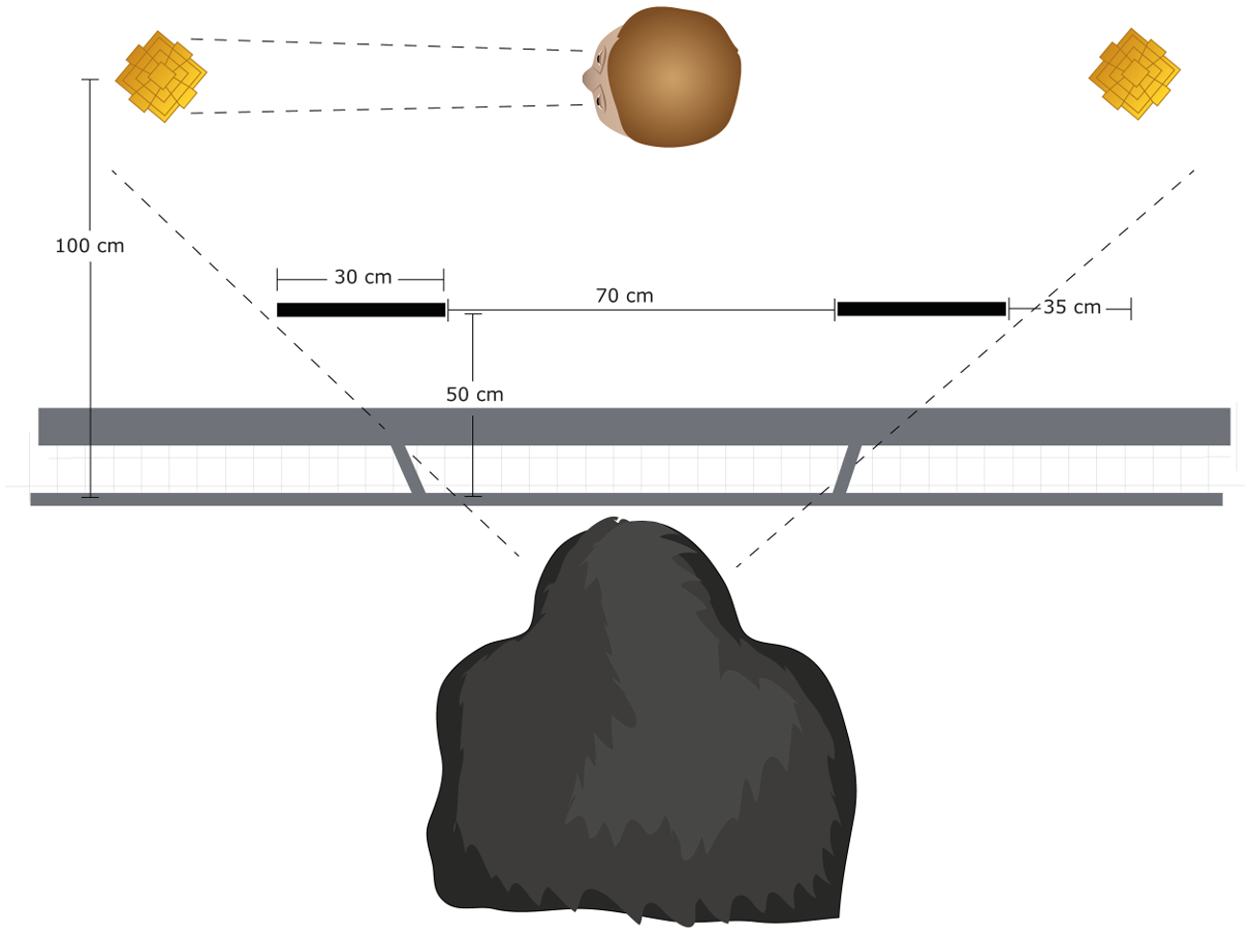
Setup for the gaze following task.

For this task, the experimenter sat in the middle between two identical objects (coloured sand moulds). From the apes’ point of view the objects were positioned behind barriers, such that the ape had to move when it intended to look at the object (see Fig 2). This is similar to the setups used in the studies by Chow et al. [16] with human infants and by Bräuer et al. [17] with great apes. Such a setup with barriers in the line of sight of the subject assures that subject’s gaze following behaviour in response to the experimenter’s gaze is unlikely to be based on an automatic response, but rather on an understanding of the gaze as referential [16, 17]. Attached to either of the two objects behind the barriers was a camera to catch the ape’s gaze when it looked at the object, which we used to score “gaze following behind barriers”. An additional camera was positioned above the head of the experimenter. This camera was used to score our equivalent to “looking in front of the barrier” in the study by Chow et al. [16]. To investigate whether an informant’s reliability affects great apes automatic response to gaze, we determined whether the ape in response to the experimenter’s gaze turned its head in the same direction and thus tried to follow his gaze, even if not moving to look behind the barrier. This was coded as “automatic gaze following”. During the gaze following task, the experimenter constantly fed the ape to keep its head in a certain position from where it could not see the objects behind the barriers. To start a trial, the experimenter stopped feeding the subject and turned his head to look at one of the objects while producing a vocalization (‘Oh!’). After holding this gaze for approximately 10 seconds, the experimenter continued to feed the subject until the start of the next trial. This was repeated for 8 trials per session for a total of 4 sessions. The gaze following task was identical in either condition.

## Analysis and results

All scoring was done using videotapes. We used two measures, “automatic gaze following” (binary; as alignment of gaze direction with that of the experimenter) as well as “gaze following behind barriers” (as number of looks to the same object the experimenter was looking at).

Automatic gaze following was coded as correct when the subject, with its first movement within the first 2 seconds (50 frames) after the onset of the experimenter’s look, aligned its gaze direction with the experimenter’s gaze direction (i.e. when it turned its head in the same direction as the experimenter did) or as incorrect when not. Four percent of the trials had to be dismissed due to opacity of the apes’ gaze direction caused by features of the enclosures.

As measure of gaze following behind barriers, any look within 10 seconds after the experimenter’s vocalisation onto the same object the experimenter was looking at was scored as correct look, looks to the other object as incorrect looks. On several occasions, the apes looked behind the barrier more than just once per trial, most of the trials though they did not move at all.

Twenty percent of all sessions were coded again by a naive observer to assess interrater reliability. Interobserver reliability for both looking behind barriers (*κ* = 0.87) as well as for automatic gaze following (*κ* = 0.8) was good. Preliminary analyses did not reveal any effects of sex or rearing history, these variables were thus collapsed for further analyses.

### Automatic gaze following

**Fig 3:**
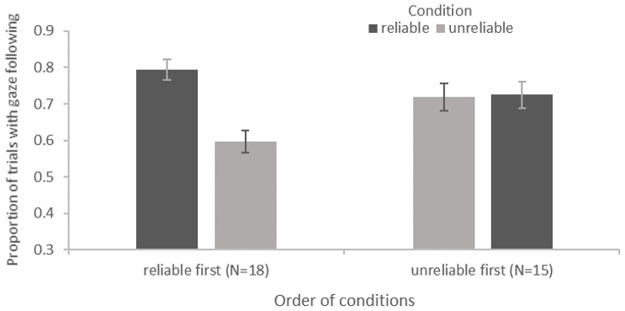
Automatic gaze following. Mean (SE) proportion of trials with correct automatic gaze following by condition and order of conditions.

As all assumptions were met, we used a repeated measures ANOVA to look at our measure of automatic gaze following. We calculated the proportion of trials with correct gaze following out of all valid trials as the dependent measure. Predictors were reliability of the experimenter, species, and order of conditions, as well as the interactions of order and species with reliability. The ANOVA revealed a main effect of reliability, *F*(1,25) = 7.526, *p* = .011, as well as an interaction effect of reliability and order of conditions, *F*(1,25) = 7.698, *p* = .010 (see Fig 3). Fig 3 shows the mean proportion of trials with automatic gaze following in the correct direction for each condition, separated in two groups depending on order of conditions.

As additional analysis before interpreting the results of the ANOVA, we examined the overall effect of reliability (i.e., the combined effect of reliability and its interactions) and order to alleviate issues with multiple testing. Therefore, we used a mixed model with Gaussian error structure to compare a full model to a null model [22]. For the full model, we used our measure for automatic gaze following as the dependent variable. Reliability of the experimenter, species, and order of conditions as well as the interactions between reliability and species, and reliability and order of conditions were included as fixed effects. Subject was included as a random effect. For the null model, reliability, its interactions, and order were removed. Using a likelihood ratio test [23], we found a significant overall effect of reliability, its interactions, and order (*χ*^2^(6) = 24.457, *p* < .001). We used the full model to ascertain that the model residuals were randomly and normally distributed as a way to confirm these assumptions of the repeated measures ANOVA. Variance inflation factors for our predictors revealed that multicollinearity was not a concern (maximum VIF = 1.003684; [24]). Model stability was assessed by removing each subject from the data, running a model on the remaining data and comparing the results to the original model based on all data. The interaction of condition and species was found to be slightly unstable. This could be due to the small sample sizes. Caution is advised regarding the results of this term.

We also compared subject’s automatic gaze following between conditions in the first session as well as in the first trial overall, however there were no significant differences.

### Gaze following behind barriers

**Fig 4:**
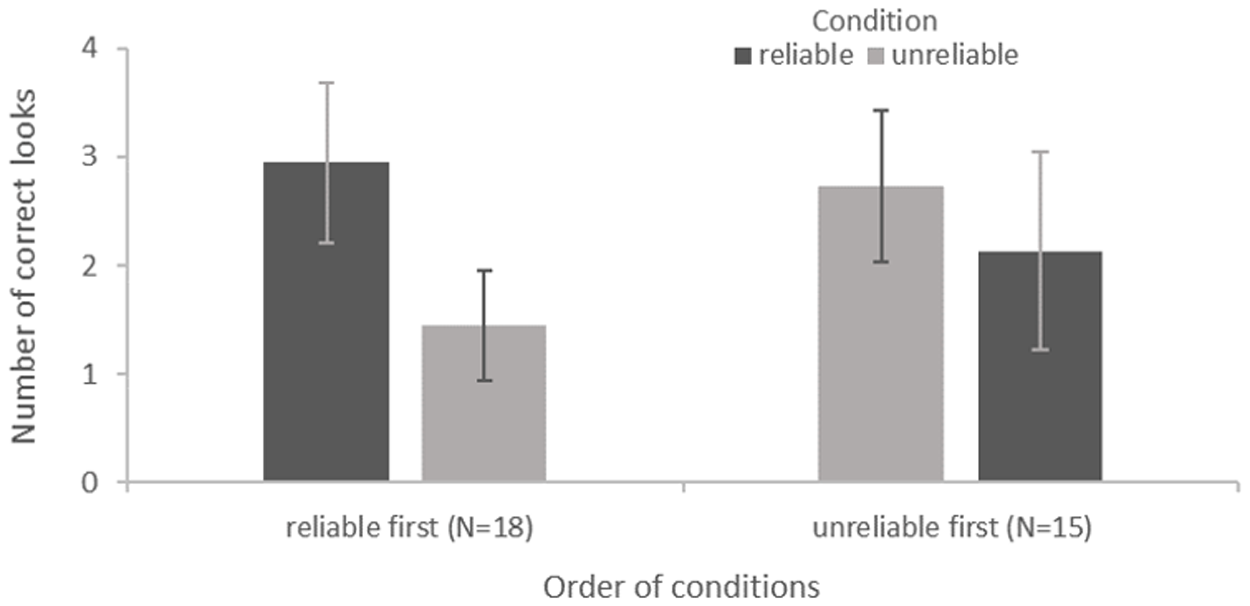
Gaze following behind barriers. Mean (SE) number of looks on the same object the experimenter was looking at by condition and order of conditions.

For our measure of gaze following behind barriers, we ran a mixed effects Poisson model as the assumptions about the distribution of residuals for a repeated measures ANOVA were not met. We used the absolute number of looks at the same object the experimenter was looking at as dependent measure. Reliability of the experimenter, species, and order of conditions as well as the interactions between reliability and species, and reliability and order of conditions were included as fixed effects. Subject was included as a random effect. After a model reduction, we found an interaction between reliability and order of conditions using a likelihood ratio test, *χ*^2^(1) = 11.7363, *p* < .001. Fig 4 shows the mean number of looks onto the same object the experimenter was looking at for each condition, separated in two groups depending on order of conditions.

In parallel to our additional analysis for our measure of automatic gaze following, we initially examined the overall effect of reliability and order to alleviate issues with multiple testing using the aforementioned mixed model with Poisson error structure and log link function. We compared the full model to a null model, which were both set up in the same way as for our measure of automatic gaze following except with our measure for gaze following behind barriers as dependent variable. We found a significant overall effect of reliability, its interactions and order of conditions (*χ*^2^(6) = 17.839, *p* < .001). Variance inflation factors for our predictors revealed that multicollinearity was not a concern (maximum VIF = 1.003684). Model stability was assessed in the same way as for the model for automatic gaze following, and the interaction of condition and species was again found to be slightly unstable. Again, this could be due to the small sample sizes. Caution is advised regarding the results of this term.

Additional analyses of subjects’ first conditions and first trials did not reach significance.

## Discussion

Animals habituate to false alarms over time to avoid exploitation and relying on false information [7]. Demonstrating a more flexible response to false information, dogs quickly cease to follow pointing gestures by an unreliable informant, while still following gestures of a naïve informant in the same task [9]. Human children show proficiency in considering the reliability of informants already at a young age. They track an informant’s reliability across different situations [15, 16] and flexibly adjust to changes in the reliability of an informant over time [8]. In search for evolutionary roots of the compelling findings on selective trust in informants in early childhood, we were interested in how our closest living relatives, great apes deal with information from previously unreliable informants. As apes consistently respond to a human’s gaze [5], we used a gaze following paradigm to investigate if that would be different depending on how reliable the information was this person offered in a previous object choice task (see also [16]). We investigated the effect of prior reliability on an automatic as well as a more explicit gaze following measure, to discern a general avoidance and a more deliberate process underlying the selectivity.

The apes in our study automatically adjusted their gaze direction in response to the experimenter’s gaze (“automatic gaze following”) according to two influencing factors: the reliability of the experimenter and order of conditions (see Fig 3). Apes who experienced the experimenter to be reliable first and unreliable later show a considerable decrease in gaze following behaviour. Apes who experienced the experimenter to be unreliable first show similarly high proportions of gaze following behaviour in both conditions. We know from previous studies on gaze following in great apes, that apes habituate to others’ gaze over trials and sessions [17, 20]. If however habituation during the gaze following task was the only influence on apes’ gaze following in our study, we would expect to see a decrease in gaze following independent of what happened in a conceptually different task previously, as the gaze task was identical in all sessions. On the other hand, if the reliability of the experimenter was the only influencing factor, we would expect to see more gaze following in the reliable condition compared to the unreliable condition. For our group of subjects that experienced the experimenter to be reliable first, both of these expectations converge and are supported by our results (see Fig 3, “reliable first”). For our group of subjects that experienced the experimenter to be unreliable first, the predictions differ. If habituation during the gaze task was the only influencing factor, we should see less gaze following in the later condition. As in the later condition the apes in this group experienced the experimenter to be reliable, prior reliability as main influence would predict gaze following to increase. In our findings, we see as the result of an interaction of both effects equally high proportions of gaze following in both conditions (see Fig 3, “unreliable first”). Both habituation during the gaze following task as well the implications of our reliability task affected great apes’ automatic gaze following behaviour. If both tasks were highly similar, these implications could stem from habituation during the reliability task [7]. However, as our object choice task was conceptually very different from our gaze following task, it seems rather unlikely that habituation in the object choice task was transferred to the gaze task. Great apes seem to employ a more flexible way of tracking the reliability of an informant than mere transferred habituation.

With our second measure of “gaze following behind barriers”, we intended to investigate whether prior reliability of the informant would also influence great apes deliberate gaze following. Here, we found apes’ gaze following behaviour to be likewise dependent on an interaction of condition and order of condition. This means, that great apes’ deliberate gaze following was similarly affected by an interaction of the effects of habituation during the gaze task and of tracking the reliability of the informant across situations. However, the relative influence of habituation during the gaze task on this measure seems to be greater. For the group that experienced the informant to be unreliable first and reliable later, we found equally high rates of “automatic gaze following”. Assuming that habituation will decrease gaze following behaviour while the influence of the reliability of the reliable informant will increase it, this suggests that the relative influence of habituation and reliability on automatic gaze following were similarly high, resulting in stable gaze following rates over time. For the same group, we now find a slight decrease in deliberate gaze following behind barriers in the later, i.e. reliable condition (see Fig 4, “unreliable first”), suggesting that the relative influence of habituation on deliberate gaze following was greater than the opposing influence of reliability. This was possibly due to the fact that in order to look behind the barriers the apes would have to move from their initial position, in spite of the fact that there was the experimenter sitting in front of them next to a bucket of food. This led to an overall quite low amount of occasions where the apes followed the gaze of the experimenter behind the barriers in either condition. Such motivational effects have already been reported by other studies on gaze following in great apes [17, 18]. Unlike some other studies on gaze following in great apes, we did not find significant differences between species [cf. 19]. Our findings suggest that tracking the reliability of an informant affects not only great apes’ automatic gaze following, but also their more deliberate gaze following behind barriers. However, an experiment with a larger sample size and hence a larger number of instances of deliberate gaze following is needed to determine this more conclusively.

Interestingly, when we tried to exclude the effects of habituation and just compare the first condition between subjects with either measure, apes who were confronted with a reliable informant were not significantly more likely to follow his gaze. Future studies could try to reduce the influence of habituation during the gaze task by using two independent experimenters as informants, similar to the original paradigms with human children (e.g. [25]).

In the study by Chow et al. [16], human infants preferably followed the gaze of a reliable experimenter behind barriers. The infants however did not differ between a reliable and an unreliable experimenter when following gaze to a target in front of the barrier. According to the authors, this rules out a general avoidance of the unreliable informant. We intended a similar comparison with our more sensitive measure for automatic gaze following and our measure of gaze following behind barriers. As we find similar results for both measures, it seems likely that apes are relying on a more general avoidance process than children that also affects implicit responses to information. However, there were slight differences between the method used by Chow et al. [16] and in our study. In our study, there was no target in front of the barrier, which could draw the attention of the subjects. Rather, the targets were still hidden behind barriers, i.e. the measure describes a different, more automatic response than our other measure, but to the same stimuli. An alternative explanation to a general avoidance of unreliable informants might therefore be that the visibility of the target stimuli in the study by Chow et al. [16] masked the effects of the (un-) reliability of the informant.

In our study, an interaction between prior reliability of the experimenter and habituation during the gaze task affected great ape’s gaze following behaviour. As habituation is context specific, this is unlikely to be caused by habituation in the previous object choice task. Rather it seems, that apes were able to implicitly infer the reliability of an informant from few interactions and apply that inference in subsequent interactions with the same informant, even in a conceptually different situation. In contrast to the findings by Chow et al. [16] with human infants, the reliability of the experimenter in our study also affected great apes’ automatic gaze response. This likely means that great apes rely on a more general avoidance process in order to refrain from responding to false information. However, further research is needed. Our study though is a first hint at the evolutionary roots of human infants’ proficiency when it comes to adjusting their trust according to an informant’s reliability.

## Supporting information

S1 Text Additional information on method. (DOCX)

S1 Data Datafile. (CSV)
