# Supplementary material for: Great apes are sensitive to prior reliability of an informant in a gaze following task
Source: PLoS One. 2017 Nov 22;12(11):e0187451. doi: 10.1371/journal.pone.0187451 (PMC5699835; doi:10.1371/journal.pone.0187451)
Supplement: S1 Text — (DOCX) [file pone.0187451.s001.docx]

**Additional information on method**

As mentioned in the paper, we used a procedure with a consistently accurate and a consistently inaccurate informant. This might seem quite artificial, as we would probably consider even an unreliable informant to be reliable sometimes. In fact, when somebody is consistently inaccurate, adult humans would probably be able to exploit this consistency by inferring the opposite of the information offered by the inaccurate informant. However, when we look at how children deal with consistent inaccuracy they seem to be unable to exploit such an informant. In a study by Pasquini, Corriveau, Koenig and Harris [23], it made no difference for 3-4 year old children whether the inaccurate informant was accurate in 0% or 25% of trials. In fact, in this study 3 year olds did not differ between a 75% and a 0% accurate informant. Certainly, it would be interesting in future research to explore great apes response to relatively inaccurate informants, but for our current study, we decided to follow procedure of the study by Chow et al. (2008) and provide the apes with a consistently inaccurate informant. In order to still control for potential exploitation of our inaccurate informant, and to ensure that the apes were paying attention and treating the experimenter as informant, we set a criterion, that the apes had to pass in their first session of either condition (otherwise the session was repeated for up to 8 times. If the criterion had not been passed in the 8^th^ session, the subject would drop out of the study). The criterion was set to choosing the cued cup in at least 6 out of 8 trials in the reliable condition, and to 2 – 6 out of 8 trials in the unreliable condition. We assumed that if the ape was paying attention and treating the experimenter as an informant, in the reliable condition it should choose the cup the experimenter pointed to in the majority of cases. Therefore, the criterion for this condition was set to 6 out of 8 trials. In the unreliable condition, the ape should not show a consistent strategy, rather they should be guessing. That means the ape should not follow the experimenter’s cues in the majority of trials, but defect at least sometimes. Therefore, the criterion in this condition was on one hand set to “following experimenter’s cues not more often than 6 out of 8 times”. On the other hand, we did not want the apes to exploit the consistent inaccuracy of the experimenter and choose the other cup in the majority of trials because we assumed that that could interfere with the experimenter’s perceived unreliability. Therefore, the criterion was on the other end set to “following the experimenter’s cues in at least 2 out of all 8 trials”, which makes the criterion “2-6 out of 8 trials” for the unreliable condition. It is worth noting, that not a single one of our subject was able to exploit the cues of the unreliable experimenter in the sense of a “reliably predictable inaccuracy”. All of them seemed to interpret the experimenter’s actions to be meant as a referential cue for the location of the food. Additionally, later in the gaze following task, there were virtually no incorrect looks, none of the apes expected the object to be in the opposite location as predicted by the informants gaze.

As the criterion was not met by three chimpanzees and one bonobo after 8 sessions, these four apes were excluded from the study. The three chimpanzees failed the criterion in the unreliable condition, as they followed the experimenter’s cues for more than 6 trials in 8 sessions consecutively. The bonobo failed the criterion in the reliable condition because she picked the right hand cup in almost every trial in 8 sessions consecutively, thus failing to follow the experimenters cues in at least 6 out of 8 trials per session. The remaining subjects passed the criterion in the reliable condition after an average of 1.73 sessions, in the unreliable condition it took them on average 1.91 sessions to pass.

**Additional reference**

1. Pasquini ES, Corriveau KH, Koenig M, Harris PL. Preschoolers monitor the relative accuracy of informants. Dev psychol. 2007; 43: 1216.
